# Comparative Review of the Algorithms for Removal of Electrocardiographic Interference from Trunk Electromyography

**DOI:** 10.3390/s20174890

**Published:** 2020-08-29

**Authors:** Lin Xu, Elisabetta Peri, Rik Vullings, Chiara Rabotti, Johannes P. Van Dijk, Massimo Mischi

**Affiliations:** 1 School of Information Science and Technology, ShanghaiTech University, Shanghai 201210, China; 2 Department of Electrical Engineering, Eindhoven University of Technology, 5600 MB Eindhoven, The Netherlands; e.peri@tue.nl (E.P.); R.Vullings@tue.nl (R.V.); dijkh@kempenhaeghe.nl (J.P.V.D.); m.mischi@tue.nl (M.M.); 3 Philips Research, 5656 AE Eindhoven, The Netherlands; Chiara.Rabotti@philips.com; 4 Clinical Physics Department at Kempenhaeghe, 6532 SZ Nijmegen, The Netherlands

**Keywords:** trunk electromyography, electrocardiography interference, template subtraction, adaptive filter, wavelet, blind source separation

## Abstract

Surface electromyogram (EMG) is a noninvasive measure of muscle electrical activity and has been widely used in a variety of applications. When recorded from the trunk, surface EMG can be contaminated by the cardiac electrical activity, i.e., the electrocardiogram (ECG). ECG may distort the desired EMG signal, complicating the extraction of reliable information from the trunk EMG. Several methods are available for ECG removal from the trunk EMG, but a comparative assessment of the performance of these methods is lacking, limiting the possibility of selecting a suitable method for specific applications. The aim of the present study is therefore to review and compare the performance of different ECG removal methods from the trunk EMG. To this end, a synthetic dataset was generated by combining in vivo EMG signals recorded on the biceps brachii and healthy or dysrhythmia ECG data from the Physionet database with a predefined signal-to-noise ratio. Gating, high-pass filtering, template subtraction, wavelet transform, adaptive filtering, and blind source separation were implemented for ECG removal. A robust measure of Kurtosis, i.e., ${\mathrm{KR}}_{2}$ and two EMG features, the average rectified value (ARV), and mean frequency (MF), were then calculated from the processed EMG signals and compared with the EMG before mixing. Our results indicate template subtraction to produce the lowest root mean square error in both ARV and MF, providing useful insight for the selection of a suitable ECG removal method.

## 1. Introduction

Surface electromyogram (EMG) is a noninvasive measure of the biopotential produced on the skin by motor unit action potentials. It is measured by placing electrodes on the skin above the muscle and has been widely used in a variety of applications. Key applications of surface EMG include the investigation of the neuromuscular system [1]; the estimation of joint loading and muscle forces [2,3]; prosthesis control [4,5,6]; and monitoring of, e.g., stress, breathing, and epilepsy [7,8,9]. In some of these applications, surface EMG is recorded from trunk muscles, e.g., diaphragm and pectoralis, and can therefore be contaminated by the electrocardiogram (ECG) [7,8,10].

In general, the EMG frequency band is between 10 and 500 Hz. An ECG signal has power up to 100 Hz [11,12], while its peak amplitude can be larger than trunk EMG [13]. Consequently, the ECG signal can distort the amplitude of the desired EMG and increase the power in the lower band of the EMG spectrum, complicating the extraction of reliable information from the trunk EMG. For instance, studies have reported that the presence of ECG interference introduces an overestimation of the absolute EMG levels and compromises the prosthesis control command [13]. The ECG should therefore be removed prior to the extraction of useful EMG features for further applications.

Several methods have been proposed for ECG removal from the trunk EMG, including gating (GT) [14], high-pass filtering (HP) [15], template subtraction (TS) [14,16], wavelet transform (WT) [10,17], adaptive filtering (AF) [18], and blind source separation (BSS) [19]. Unfortunately, each method has its own limitation. For instance, GT inevitably results in a loss of useful EMG data. TS is based on the assumption that the EMG is Gaussian-distributed with zero mean, which may be not fully satisfied in some conditions. HP cannot remove all the ECG spectrum while canceling the low-frequency EMG components. In addition, the determination of the optimal cutoff frequency is challenging. For AF, an additional sensor measuring a clean ECG reference is usually required [18]. For both WT and BSS, automatic identification of the noise components is difficult.

Considering the limitations of the available ECG removal methods, a complete comparison of the performance of different methods may provide useful insight for the selection of the most appropriate ECG removal method in a specific application. Indeed, several studies have compared the performance of different ECG removal methods [10,20,21]. However, the comparison in these studies is incomplete, including only a subset of the available methods. For instance, only TS and HP were employed in [20]. Zhou et al. investigated the performance of TS, WT, and AF for prosthesis control [10], and Willigenburg et al. compared the performance of HP, AF, and independent component analysis (ICA) on ECG interference removal [21].

It is clear that the available methods for ECG removal from the trunk EMG have limitations, and a complete comparison of the performance of these methods using the same dataset is missing. The aim of the present study is therefore to review the available ECG removal methods from the trunk EMG and compare their performance using the same dataset. To this end, in vivo EMG signals recorded on the biceps brachii are employed to mimic the trunk EMG, and ECG data from the Physionet database are used to mimic the ECG interference. A synthesis dataset is then generated by combining these two signals. By use of the realized synthetic dataset, the ground truth EMG and ECG signals are available, which enable us to accurately benchmark different algorithms for ECG removal.

For performance evaluation, a robust measure of Kurtosis, i.e., ${\mathrm{KR}}_{2}$, and two EMG features—the average rectified value (ARV) and mean frequency (MF)—are calculated from the EMG signals obtained by different ECG removal methods and then compared with that derived from the EMG before mixing. Kurtosis is a fourth-order statistics indicating the non-Gaussianity, and it is used to measure the peakedness of a distribution. ${\mathrm{KR}}_{2}$ is a robust measure of Kurtosis and has been suggested as a good indicator of ECG interference in an EMG signal [22]; a higher ${\mathrm{KR}}_{2}$ indicates more ECG interference [22]. ARV is a measure of the EMG amplitude and is widely used for myoelectric prosthesis control [10]. MF is calculated as the first statistical moment of the EMG amplitude spectrum and is usually used as an indicator of myoelectric fatigue [23]. Both ARV and MF are common EMG features and are therefore considered as performance measures for different ECG removal methods, in line with the work in [20].

## 2. Methods

### 2.1. Data Collection and Preprocessing

#### 2.1.1. EMG Data Collection

Surface EMG was recorded on the biceps brachii from ten right-handed healthy college students (9 males and 1 female; age: 23–28 years), who were asked to sign a written informed consent prior to participation in the study. The test procedures were approved by the ethical committee of the Máxima Medical Center (MMC, Veldhoven, The Netherlands).

**Figure 1:**
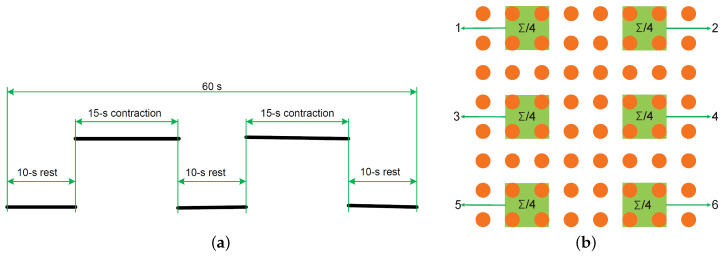
(**a**) Scheme of the measurement protocol. (**b**) Six subsets of electrodes employed to generate six electromyogram (EMG) signals.

The experimental set-up described in [24] was employed to generate a constant force pulling down a rope connected to the subject’s dominant arm through a handle. The subject was instructed to pull against the rope in order to perform isometric arm exercise with the back straight and the elbow angle equal to 90 degrees. The applied force was 50% of his/her maximum voluntary contraction (MVC), which was measured prior to the exercise by a load-cell embedded in the experimental setup following the protocol described in [25]. The entire experiment took 60 s, including two 15 s isometric contractions and three 10 s rest periods, as shown in Figure 1a.

#### 2.1.2. ECG Interference

Sixty-second surface EMG was recorded on the biceps by an 8×8 high-density electrode grid aligned along the muscle fiber direction. The diameter of each electrode is 4 mm and the interelectrode distance is 8 mm. A circular (1 cm diameter) Ag/AgCl electrode was placed on the subject’s right clavicle as ground. A Refa amplifier (TMS International, Enschede, The Netherlands) was adopted to amplify and digitize the recorded signal at a sampling frequency of 2048 Hz.

Twenty ECG datasets were randomly selected from the PhysioNet PTB Diagnostic ECG Database, including ten healthy ECG signals ( s0467_re, s0468_re, s0471_re, s0472_re, s0473_re, s0474_re, s0478_re, s0479_re, s0481_re, and s0490_re) and ten dysrhythmia ECG signals ( s0207_re, s0349lre, s0018lre, s0393lre, s0391lre, s0338lre, s0366lre, s0440_re, s0494_re, and s0546_re). Goldberger, A.L., et al. [26]. These pathological ECG signals consist of different heart diseases, such as congenital complete AV block, atrial fibrillation, coronary artery disease, palpitation, and WPW syndrome. Each dataset contained 15 simultaneously measured signals, including the conventional 12 leads (I, II, III, aVR, aVL, aVF, and V1–V6) and the 3 Frank lead ECGs (VX, VY, and VZ). The length of each recording was 60 seconds and the sampling frequency 1000 Hz. The six precordial leads (V1–V6) were measured on the chest with respect to the Wilson’s central terminal, and were therefore adopted to mimic ECG interference in trunk EMG measurements.

#### 2.1.3. Synthetic Trunk EMG and Preprocessing

Six EMG signals, in line with the number of ECG channels, were extracted from the 8×8 high-density electrode grid for each subject. Each EMG signal was generated by taking the average of a subset of four channels, as shown in Figure 1b. The extracted EMG signals were resampled at 1000 Hz in order to obtain the same sampling frequency as the ECG signals.

In general, a frequency band between 10 and 500 Hz is considered for a EMG signal [11]. Therefore, the first step for analysing a trunk EMG in real practice is bandpass filtering. Consequently, part of the ECG interference in the trunk EMG is also filtered. In the present study when generating the data, the bandpassing filtering (10–500 Hz) was considered before mixing, which has the same effects as applied to the mixture but provides benchmark to evaluate the performance of different ECG removal methods. Furthermore, as all signals were resampled at 1000 Hz, only a 10 Hz high-pass filter was implemented by a third-order Butterworth filter.

A second-order infinite impulse response filter was applied to both signals in order to remove possible power-line interference. Beyond, a third-order Butterworth filter with cutoff of 200 Hz was applied to the ECG signals in order to remove high-frequency instrumentation noise, avoiding unrealistic noise crosstalk between the EMG and ECG signals.

**Figure 2:**
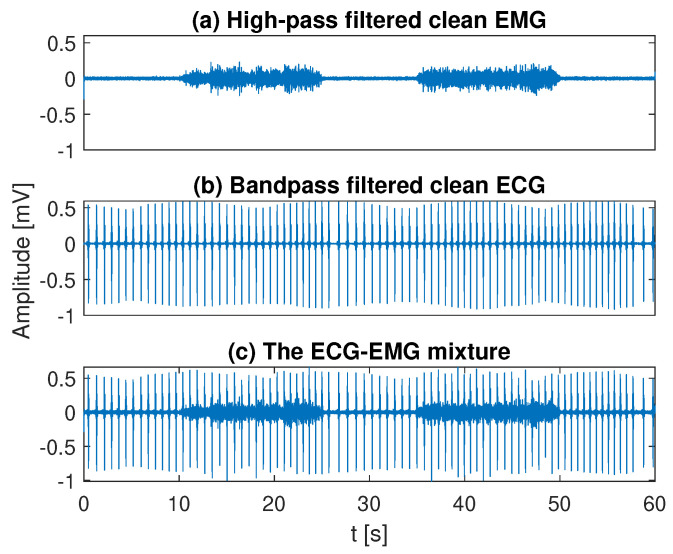
An example of the EMG (**a**), ECG (**b**), and their mixture (**c**).

Next, the EMG signals were rescaled in order to obtain a realistic EMG-ECG signal-to-noise ratio (SNR), i.e., −10 dB, realistic for trunk EMG [10]. Finally, the preprocessed EMG and ECG signals were added together, producing six mixtures for each subject mimicking six channels with trunk EMG contaminated by ECG interference, as given by

(1) $$\underline{x}\left(k\right)=\underline{m}\left(k\right)+\underline{c}\left(k\right),$$

(2) $$\underline{x}\left(k\right)={\left[x_{1}\left(k\right),\;x_{2}\left(k\right),\;\ldots ,\;x_{6}\left(k\right)\right]}^{T},$$

(3) $$\underline{m}\left(k\right)={\left[m_{1}\left(k\right),\;m_{2}\left(k\right),\;\ldots ,\;m_{6}\left(k\right)\right]}^{T},$$

(4) $$\underline{c}\left(k\right)={\left[c_{1}\left(k\right),\;c_{2}\left(k\right),\;\ldots ,\;c_{6}\left(k\right)\right]}^{T},$$

where *k* is the discrete time and $\underline{x}\left(k\right)$, $\underline{m}\left(k\right)$, and $\underline{c}\left(k\right)$ represent the EMG-ECG mixture, the EMG after preprocessing, and the ECG after preprocessing, respectively. An example of these three signals is shown in Figure 2. Ten healthy ECG signals and ten dysrhythmia ECG signals were considered in the present study, generating two categories of mixtures, EMG contaminated by healthy ECG and EMG contaminated by dysrhythmia ECG, respectively.

### 2.2. ECG Interference Removal

#### 2.2.1. Gating

The gating method implemented in the present study was the one described in [14]. The idea was to set samples to zero in the QRS time interval, which is normally about 0.1 s, to zero. To this end, for each channel *j* (*j* = 1, 2, …, 6), the position of the *i*th R-peak, $t_{ji}$, was detected by a low complexity algorithm described in [27]. Considering that the duration of the ECG waveform may be influenced by variations in the R-R interval, $t_{RR}$, of different subjects, the QRS time interval of the *i*th R-peak was determined as [($t_{ji}$−$0.05t_{RR}$) ($t_{ji}$ + $0.05t_{RR}$)]. Finally, the samples in the identified QRS interval were set to zero.

#### 2.2.2. High-Pass Filtering

In previous studies, a finite impulse response (FIR) filter with a 820-coefficient Hamming window was employed to realize a high-pass filter for ECG removal [15]. A recent study suggested a 4th-order Butterworth filter to perform better than a FIR filter [20]. Consequently, the Butterworth filter adopted in was implemented in the present study. As the performance of high-pass filtering methods can be influenced by the cutoff frequency, five cutoff frequencies: 20, 30, 40, 50, and 60 Hz, were separately implemented and tested in the present study, in line with the work in [20].

The two EMG features, i.e., ARV and MF, used for performance evaluation (Section 2.3) were calculated from the reconstructed EMG and the EMG before mixing. The root mean square error (RMSE) in these two EMG features was then calculated as a criterion to identify the optimal cutoff frequency.

#### 2.2.3. Template Subtraction

The ECG template subtraction method takes advantage of the quasi-periodic characteristics of the ECG signal, and is based on the assumption that the EMG has a zero mean Gaussian distribution [10,16]. Similar to gating, the first step in template subtraction was to detect the position of the R peaks in each channel. The full ECG waveform, including the P wave, QRS, and T wave, was then considered in order to build an ECG template. In general, the P wave, QRS complex, and T wave durations are approximately 0.20 s, 0.1 s, and 0.40 s, respectively [28].

By taking the R–R interval into consideration, the full ECG waveform of the $i_{th}$ R-peak in channel *j*, ECG${}_{ji}$, was then determined as the samples in the interval [($t_{ji}\;-\;0.25t_{RR}$) ($t_{ji}\;+\;0.45t_{RR}$)],

(5) $${\mathrm{ECG}}_{ji}=x_{j}\left[\left(t_{ji}-0.25t_{RR}\right)\;\left(t_{ji}+0.45t_{RR}\right)\right].$$

An ECG template for the *i*th R-peak, ${\hat{TP}}_{ji}$, was then estimated by taking the average of 11 neighboring ECG waveforms, as given by

(6) $${\hat{\mathrm{TP}}}_{ji}=\left\{\begin{array}{cc} \frac{1}{11}\sum_{y=1}^{11}{\mathrm{ECG}}_{jy}, & i\leq 5\\ \frac{1}{11}\sum_{y=i-5}^{i+5}{\mathrm{ECG}}_{jy}, & 5<i\leq Y-5\\ \frac{1}{11}\sum_{y=N-10}^{N}{\mathrm{ECG}}_{jy}, & i>Y-5 \end{array}\right.$$

where *Y* is the number of the detected R peaks. The generated template ${\hat{\mathrm{TP}}}_{ji}$ was scaled by a factor $\mu_{ji}$ and subtracted from ${\mathrm{ECG}}_{ji}$. The scaling factor $\mu_{ji}$ was determined such that the mean square error (MSE) ${\mathrm{ECG}}_{ji}\;-\;\mu_{ji}\cdot {\hat{\mathrm{TP}}}_{ji}^{2}$ is minimized.

#### 2.2.4. Wavelet Transform

**Figure 3:**
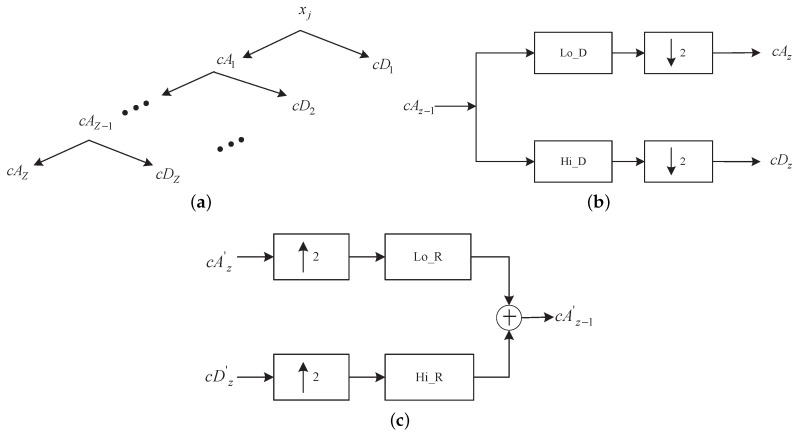
Schematic of wavelet decomposition. j=0,…,J−1 indicates the decomposition level and $cA_{0}=x_{j}$. (**a**) Structure of the J level decomposition tree; (**b**) operation in each level to decompose the signal; (**c**) operation in each level to reconstruct the signal.

Wavelet transform has been suggested for ECG interference removal by previous studies [10,17], and was therefore investigated in the present study. The wavelet decomposition was calculated using Mallat pyramid algorithm [29]. For each channel *j*, the EMG–ECG mixture $x_{j}$ was decomposed into Z levels of detail coefficients, $cD_{1}$, $cD_{2}$, …, $cD_{Z}$, and one approximation coefficient, $cA_{Z}$, as shown in Figure 3a. The approximation coefficient at each decomposition level, $cA_{z}$ (*z* = 1, 2, …, *Z*), was obtained by convolving the approximation coefficient of the previous level, $cA_{z-1}$ ($cA_{0}=x_{j}$), with a low-pass decomposition filter, $\mathrm{Lo}\underline{\;\;}D$, followed by a factor-2 downsampling. The detail coefficients $cD_{z}$ were obtained by convolving $cA_{z-1}$ with a high-pass decomposition filter $\mathrm{Hi}\underline{\;\;}D$ followed by the downsampling, as shown in Figure 3b. The high-pass and low-pass decomposition filters were derived from a mother wavelet function and designed to form quadrature mirror filters. The db4 mother wavelet was reported to produce the best performance for ECG interference removal in diaphragm EMG, and was therefore adopted as the basic wavelet function in the present study [17].

Seven-level wavelet decomposition was implemented, in line with [10]. A nonlinear thresholding process as described in [10] was applied to the high-scale low-frequency components: $cA_{7}$, $cD_{7}$, $cD_{6}$, and $cD_{5}$, in order to remove the ECG interference, as suggested in [10]. The nonlinear thresholding process was implemented such that the samples in the coefficients higher than a certain threshold were set to zero. The threshold was dynamically calculated by multiplying a gain *G* with the average value of neighboring samples.

The inverse wavelet transform was then performed using the coefficients after thresholding in order to obtain the EMG signal with most of the ECG interference removed. As shown in Figure 3c, starting from the highest level, the approximation and detail coefficients, $cA_{z}^{\prime }$ and $cD_{z}^{\prime }$, were upsampled by a factor of 2, and then filtered with the reconstruction low-pass and high-pass filters, $\mathrm{Lo}\underline{\;\;}R$ and $\mathrm{Hi}\underline{\;\;}R$, respectively. The reconstruction filter pair was derived from the db4 wavelet function, the same as the decomposition filter pair. The outputs of the reconstruction filters were then added together in order to obtain the approximation coefficient for the next lower level, $cA_{z-1}^{\prime }$. By recursively applying the procedure until reaching the lowest level, the inverse wavelet transform was obtained, producing a reconstruction of the EMG signal free of ECG interference, $\hat{m_{j}}\left(k\right)$.

#### 2.2.5. Adaptive Filtering

Adaptive filtering has been widely used for noise reduction in biomedical applications [30,31,32,33]. In general, an adaptive filter estimates the transfer function between a noise reference signal and the actual noise affecting the signal; any noise correlated to the reference signal is then removed from the raw signal [34]. The reference signal that is correlated with the noise contaminating the signal of interest is, therefore, of key importance for an adaptive filter.

**Figure 4:**
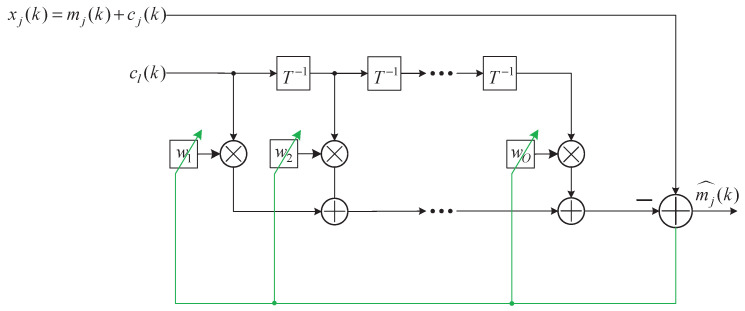
Structure of the adaptive filter. $x_{j}\left(k\right)$ is the EMG–ECG mixture in the *j*th channel, $c_{l}\left(k\right)$ is one of the nine ECG leads in the same dataset as $c_{j}\left(k\right)$ but was not used for mixing, $T^{-1}$ indicates a one-sample delay, and *O* is the filter order.

The structure of the adaptive filter implemented in the present study is shown in Figure 4, where $x_{j}\left(k\right)$ is the mixture, $c_{l}\left(k\right)$ is a reference containing only ECG signal, $T^{-1}$ stands for a delay of one sample, and *O* is the filter order. The reference signal $c_{l}\left(k\right)$ was weighted by the coefficients of the adaptive filter, $w_{1}$ to $w_{O}$, and then subtracted from the EMG-ECG mixture. The coefficients of the adaptive filter can be obtained by minimizing the squared residual

(7) $$\hat{m_{j}}{\left(k\right)}^{2}=x_{j}\left(k\right)-\underline{c_{l}}\left(k\right)\underline{W}{\left(k\right)}^{2},$$

where $\underline{c_{l}}\left(k\right)$ was a vector $\left[c_{l}\left(k\right)\;c_{l}\left(k-1\right)\;\ldots \;c_{l}\left(k-O+1\right)\right]$ and $\underline{W}\left(k\right)$ was ${\left[w_{1}\;w_{2}\;\ldots \;w_{O}\right]}^{T}$. A normalized least mean square (NLMS) algorithm was implemented to calculate $\underline{W}\left(k\right)$ [32], given as

(8) $$\begin{array}{cc} \underline{W}\left(k+1\right) & =\underline{W}\left(k\right)+\mu \cdot {\underline{c_{l}}}^{T}\left(k\right)\cdot \hat{m_{j}}\left(k\right)/\sigma^{2}, \end{array}$$

(9) $$\begin{array}{cc} \sigma^{2} & =\underline{c_{l}}\left(k\right)\cdot {\underline{c_{l}}}^{T}\left(k\right)/O+\varepsilon , \end{array}$$

where μ was the step size controlling the stability and the convergence rate to the optimal solution and ε a small constant to avoid σ to be zero.

In real applications, the performance of the adaptive filter may be affected by the position of the electrode recording the reference ECG as well as the filter order. Therefore, for each EMG–ECG mixture, the nine ECG leads in the PTB database that were not used for mixing were separately implemented as a reference in the present study. Besides, 10 filter orders, i.e., from 1 to 20 in steps of 2, were also implemented. A robust measure of Kurtosis, i.e., ${\mathrm{KR}}_{2}$, which has been suggested as a good indicator of ECG interference in an EMG signal [22], was calculated from the adaptively filtered signals. For each ECG–EMG mixture, the reference-order combination that produced the lowest ${\mathrm{KR}}_{2}$ was adopted for the comparison with other ECG removal algorithms.

#### 2.2.6. Blind Source Separation

Blind source separation, particularly ICA, is a powerful tool to extract signal from noise measurements and has been employed for ECG interference removal from trunk EMG by previous studies [19,35]. By assuming the EMG and ECG signals to be independent, the ICA approach can be formulated as

(10) x=As,

where x is the observed six-channel EMG-ECG mixtures; s is a *m*-dimensional random vector with its components representing different independent sources, e.g., EMG, ECG, and other noise; and A is the 6×m mixing matrix. The number of independent sources *m* is assumed to be less than the number of channels. The objective of ICA is to estimate the mixing matrix A and thus reconstruct the independent sources by

(11) $$s=A^{-1}x.$$

In the present study, the fixed-point fast ICA described in [36] was implemented in order to estimate the mixing matrix A and thus decompose the EMG-ECG mixtures into different sources. The ICA components representing ECG signals were then identified. To this end, a third-order Butterworth low-pass filter with cutoff at 30 Hz was applied to one of the six EMG-ECG mixtures, $x_{j}$, producing an approximate estimate of the ECG interference. The correlation coefficient between each ICA component and the approximate ECG estimate was then calculated. The two ICA components with the highest correlation coefficients were considered as ECG source and set to zeros. The modified ICA components were then multiplied by the mixing matrix A in order to reconstruct six-channel EMG with most ECG interference removed.

### 2.3. Evaluation

To compare the performance of different algorithms, a statistical metric, ${\mathrm{KR}}_{2}$, which is a robust measure of Kurtosis, was first calculated as a measure of ECG interference in the EMG signals, as given by [22]

(12) $${\mathrm{KR}}_{2}=\frac{F^{-1}\left(0.975\right)-F^{-1}\left(0.025\right)}{F^{-1}\left(0.75\right)-F^{-1}\left(0.25\right)}-2.91,$$

where $F^{-1}$ is the inverse cumulative distribution function of the data. If the data is normally distributed, we obtain $F^{-1}\left(0.975\right)-F^{-1}\left(0.025\right)=1.96$ and $F^{-1}\left(0.75\right)-F^{-1}\left(0.25\right)=0.6745$. Thus, ${\mathrm{KR}}_{2}$ is zero if the data have standard Gaussian distribution.

In addition, two most frequently used EMG features—ARV and MF—were also calculated for performance assessment. ARV was defined as

(13) $$\mathrm{ARV}=\frac{1}{N}\sum_{k=1}^{N}\hat{m_{j}}\left(k\right),$$

where $\hat{m_{j}}\left(k\right)$ is the reconstructed EMG and N the number of samples. For MF, the short-time Fourier transform (STFT) of $\hat{m_{j}}\left(k\right)$ was first calculated. MF was then calculated as the first statistical moment of the STFT amplitude spectrum between 10 and 500 Hz, as given by

(14) $$\mathrm{MF}=\frac{\sum_{n=N_{1}}^{N_{2}}f_{n}\cdot S_{n}}{\sum_{n=N_{1}}^{N_{2}}S_{n}},$$

where *n* is the STFT point, and $f_{n}$ and $S_{n}$ the frequency and amplitude spectrum of the EMG at *n*, respectively. The lower and upper limits of *n*, $N_{1}$, and $N_{2}$ were designed such that the corresponding frequencies were 10 and 500 Hz, respectively.

For each channel, ${\mathrm{KR}}_{2}$ was calculated on the entire 60 s reconstructed EMG signal. ARV was calculated in a 1 s sliding window with no overlap, in line with [25]. MF was calculated using the same sliding window, but only during the two contraction periods as shown in Figure 1a. Moreover, the three evaluation metrics were also calculated from the same EMG channel before mixing. For each metric, the RMSE between the values estimated from the reconstructed EMG and that derived from the EMG before mixing was then calculated in each channel. As only one ${\mathrm{KR}}_{2}$ value was derived for each channel, the estimated RMSE corresponds to the absolute error. However, in order to keep in line with the other two metrics, we would use RMSE for ${\mathrm{KR}}_{2}$ in the rest of this paper.

For each evaluation metric, 60 RMSEs obtained from the ten subjects with each consisting of six channels were adopted for the comparison between different algorithms. Based on the one-sample Kolmogorov–Smirnov test, data were not normally distributed. Consequently, a nonparametric Friedman test was employed to test the difference of all algorithms globally. In addition, a post hoc test using Bonferroni’s method was employed to test the difference between each two algorithms.

Moreover, to evaluate the computational complexity, the execution time of different methods was determined and then compared. All the analysis was implemented in Matlab${}^{®}$ 2019a (MathWorks, Natick, MA, USA). The adopted computer was a DELL TECH-20191012RQ PC (Dell, Round Rock, TX, USA), with an Inter${}^{®}$ Core™) i9-9900K CPU @ 3.60 GHz (16 CPUs) processor (Inter, Santa Clara, CA, USA).

## 3. Results

For high-pass filtering, our results in Table 1 show 40 Hz to produce the lowest RMSE for the ARV estimation and 30 Hz to produce the lowest RMSE for the MF estimation. Taking both ARV and MF into account, 30 Hz was considered as the optimal cutoff frequency and was implemented for the comparison with other ECG-removal methods.

**Figure 5:**
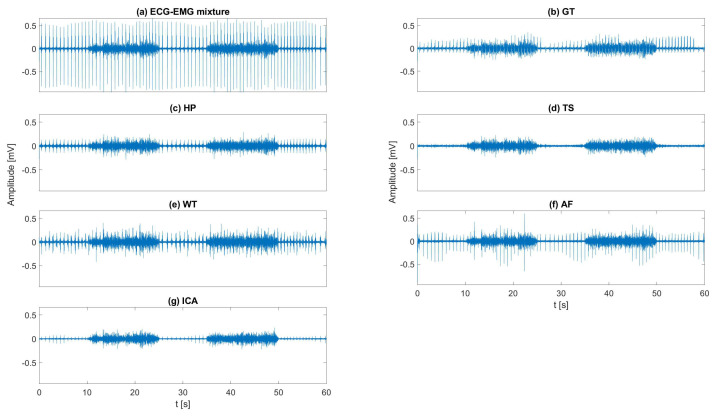
Example of ECG removal: (**a**) ECG-EMG mixture; (**b**) EMG reconstructed by GT; (**c**) EMG reconstructed by HP; (**d**) EMG reconstructed by TS; (**e**) EMG reconstructed by WT; (**f**) EMG reconstructed by AF; (**g**) EMG reconstructed by ICA.

Figure 5 shows an example of the estimated EMG signals by different ECG removal methods applied to the EMG–ECG mixture with healthy ECG. After implementing the ECG removal algorithms, ECG residuals are clearly recognizable for GT, HP, WT, and AF. Considering two components as ECG interference, ICA seems to produce less residuals but reduced EMG amplitude (Figure 5g). Almost no residuals are observed in the signals obtained by TS (Figure 5d).

**Figure 6:**
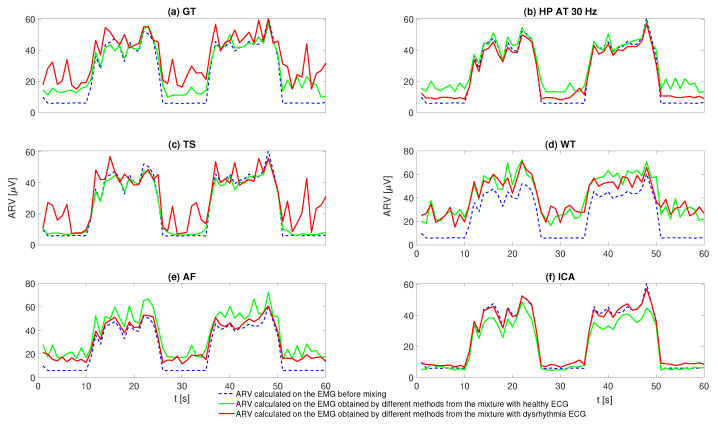
Example of the ARV results calculated from the EMG signals obtained by different ECG removal methods: (**a**) ARV results by GT; (**b**) ARV results by HP; (**c**) ARV results by TS; (**d**) ARV results by WT; (**e**) ARV results by AF; (**f**) ARV results by ICA.

A representative example of ARV calculated in 1 s sliding window with no overlap is shown in Figure 6. For the EMG–ECG mixture with healthy ECG (solid green line), GT, HP, and AF produce overestimates in ARV due to the ECG residuals, especially during the rest periods. ICA produces no overestimates during the rest periods, but underestimates during the contraction periods. Given the threshold adopted in this study, WT seems to produce overestimates for both rest and contraction periods. In contrast, ARVs calculated from the EMG signals obtained by TS match the reference values, as shown in Figure 6c. Similar trend is observed in the EMG-ECG mixture with dysrhythmia ECG (solid red line). However, the performance of several methods, particularly TS, seems to degrade.

**Figure 7:**
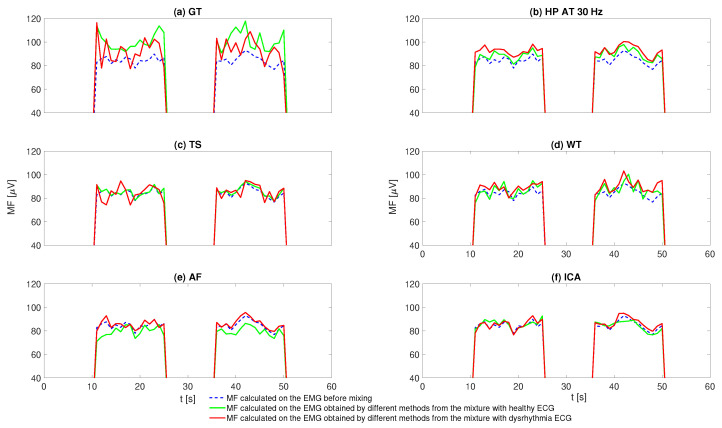
Example of the MF results calculated from the EMG signals obtained by different ECG removal methods: (**a**) MF results by GT; (**b**) MF results by HP; (**c**) MF results by TS; (**d**) MF results by WT; (**e**) MF results by AF; (**f**) MF results by ICA.

Figure 7 shows a representative example of the MF results calculated using the same sliding window as used for ARV, but applied during the contraction periods only. For the EMG–ECG mixture with healthy ECG (solid green line), overestimates in MF can be observed with GT and HP while underestimates are obtained with AF. TS produces the best MF results. Data with dysrhythmia ECG (solid red line) show a similar trend. However, AF produces much less underestimation, while TS performs slightly worse as compared with data with healthy ECG.

**Figure 8:**
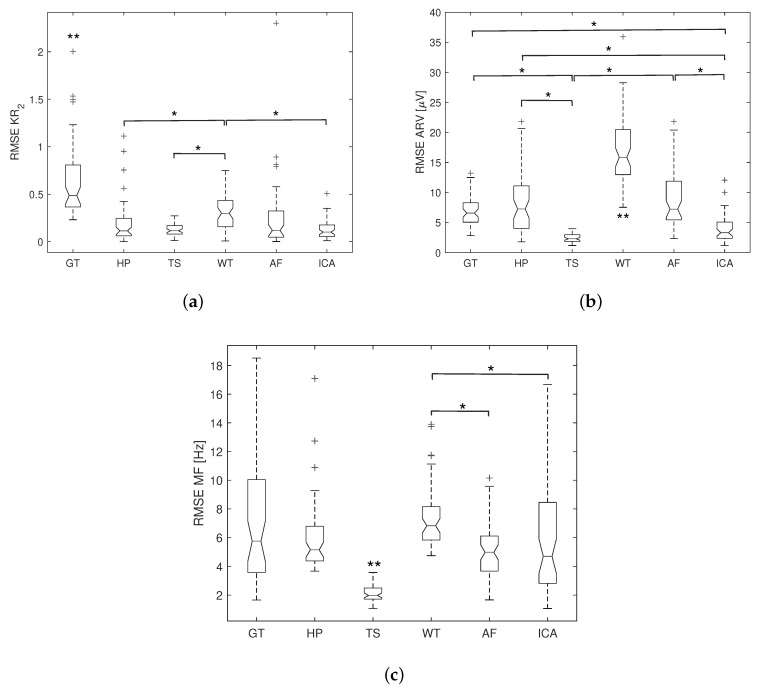
Boxplot of the RMSE results for different ECG removal methods with healthy ECG interference: (**a**) RMSE in ${\mathrm{KR}}_{2}$; (**b**) RMSE in ARV; (**c**) RMSE in MF. Data are presented as median, the first and third quartiles, and the upper and lower adjacent. ${}^{+}$ indicates the outliers (whisker length = 1.5). ** indicates significant (p<0.05) difference from all other methods, and * indicates significant (p<0.05) difference between two methods.

**Figure 9:**
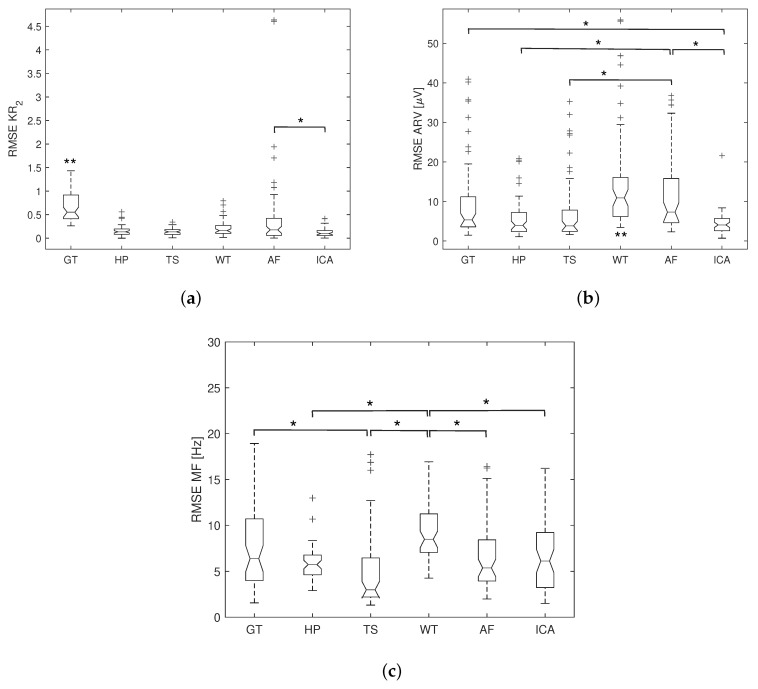
Boxplot of the RMSE results for different ECG removal methods with dysrhythmia ECG interference: (**a**) RMSE in ${\mathrm{KR}}_{2}$; (**b**) RMSE in ARV; (**c**) RMSE in MF. Data are presented as median, the first and third quartiles, and the upper and lower adjacent. ${}^{+}$ indicates the outliers (whisker length = 1.5). ** indicates significant (p<0.05) difference from all other methods, and * indicates significant (p<0.05) difference between two methods.

The RMSE results of KR${}_{2}$, ARV, and MF over all subjects are numerically reported in Table 2 and graphically demonstrated in Figure 8 and Figure 9. Data are presented as median and interquartile value as they are not normally distributed. For both healthy and dysrhythmia ECG interference, the highest RMSE in KR${}_{2}$ is observed with GT and the lowest with ICA. The KR${}_{2}$ RMSE for TS is slightly higher than that of ICA. The statistical significance is indicated by * and ** in Figure 8 and Figure 9. WT produces the largest RMSE, while TS produces the smallest RMSE in ARV and MF for both datasets. However, for the data with dysrhythmia ECG interference, all methods produce larger RMSE in KR${}_{2}$ and MF, and TS, AF, ICA produce larger RMSE in ARV, while other methods yield smaller ARV RMSE.

Finally, the average execution time of different algorithms for one dataset consisting of six channels is shown in Table 3. It is clear that AF requires the highest execution time while GT requires the lowest execution time. The execution time of TS is slightly higher than ICA.

## 4. Discussion

Six different algorithms for ECG removal from trunk EMG were implemented and compared in the present study. Although all these algorithms are available in the literature, no study presents a complete comparison of all of them. The novelty of the present study lies, therefore, in the comparison of all these algorithms based on the same dataset. Synthetic data were generated by combining in vivo EMG from the biceps and ECG from the chest, enabling quantitative evaluation of the performance of these algorithms as the ground truth was known. In addition, both healthy and dysrhythmia ECG interference were considered in the present study, providing relevant indications on the robustness of the algorithms to non-physiological ECG rhythms. For evaluation, ${\mathrm{KR}}_{2}$ was adopted as a statistical measure of the ECG interference in the reconstructed EMG. Moreover, ARV and MF are common EMG features which have been widely used in many applications [10]; therefore, they were employed to assess the performance of the different algorithms.

Gating is the simplest method, as indicated by the execution time in Table 3. However, it inevitably results in a loss of EMG information. In order to restrict this information loss, a short gating window, i.e., 0.1 s, is adopted in the present study, in line with previous studies [27]. Such a short window, on the other hand, results in ECG residuals in the processed EMG signal, producing overestimates in the ARV, in particular during the rest periods. A balance between information loss and ECG residual should be considered when using gating for ECG removal, which is rather challenging.

For high-pass filtering method, a 4th-order Butterworth filter with five different cutoff frequencies was implemented in the present study [20]. Our results indicate 30 Hz cutoff to produce the best results, in line with the work in [20]. However, Zhou et al. reported that cutoff frequencies between 50 and 90 Hz produce the most suitable signals for myoelectric prosthesis control [10]. This discrepancy may be explained by the exercise type (isodynamic) and performance criteria (ARV only) adopted in [10]. Furthermore, due to spectrum overlap, the implemented high-pass filter suppresses some useful EMG information while leaving some ECG residuals in the processed signal, leading to an overestimation in the ARV during the rest periods.

For the adaptive filtering method, a reference signal correlated with the ECG interference is usually required [32]. In the present study, the optimal reference channel was identified from the nine ECG leads that were not used for mixing. However, there are still considerable ECG residuals in the best filtered EMG signal, resulting in the large RMSE in both ARV and MF. Moreover, the execution time of the adaptive filtering method is much longer than all other methods.

The wavelet transform method based on nonlinear thresholding was used for ECG removal in prosthesis control by previous authors [10], and was therefore implemented and compared in the present study. Our results show WT to produce overestimates in ARV due to the ECG residuals, indicating the adopted threshold to be too low. An increased threshold may lead to reduced residuals. However, this can also remove EMG information. The same challenge stands for the ICA method. In the present study, two out of six ICA components were set to zero in order to remove the ECG interference, producing relatively low ECG residuals. However, some EMG information was also removed, as evidenced by the reduced EMG amplitude (Figure 5g) and the underestimate in ARV (Figure 6f). Optimization of the WT and ICA methods is also challenging in real applications, where the ground truth is normally unknown.

The template subtraction method utilizes the quasi-periodic characteristics of the ECG signal and assumes the EMG to be Gaussian distributed with zero mean. Our results suggest TS to produce the lowest RMSE in ARV and MF and the second lowest RMSE in KR${}_{2}$ among all the six methods, irrespective of the data consisting of healthy or dysrhythmia ECG interference. Besides, different from ICA, TS can work on single channel recording rather than multichannel recordings. Furthermore, it does not need a clean ECG as a reference, which instead is required by the adaptive filtering method. Therefore, TS seems to be the most suitable method for ECG removal in trunk EMG measurements although its computational time is slightly higher than GT, HP, WT, and ICA.

It should be noticed that, in the present study, the synthetic data is generated by real-life EMG and ECG recorded by physical systems, which contain the instrumentation noise and, possibly, the power-line interference. Therefore, a notch filter was employed in the preprocessing in order to remove the power-line interference. In addition, a 200 Hz low-pass filter was implemented for the ECG before mixing in order to reduce the high-frequency instrumentation noise and, therefore, reduce the crosstalk between the noise from the two signals. Other noise, such as the motion artifacts, is not considered since it is out of the scope of the present study.

It should also be noticed that, the generated synthetic data has a fixed SNR, i.e., −10 dB, which is realistic for trunk EMG [10]. However, although not all the six methods were compared, Zhou et al. [10] and Drake et al. [20] suggested that the optimal ECG removal method may depend on movement type and contraction level. Therefore, dedicated studies focused on investigating the influence of movement type and contraction level on the performance of all these six ECG removal methods are still needed in the future.

## 5. Conclusions

In the present study, six ECG removal algorithms for trunk EMG measurements were implemented and compared for the first time using synthetic data generated by combining in vivo bicep EMG and healthy or dysrhythmia ECG recorded on the chest. In order to assess the performance of the ECG removal methods, ${\mathrm{KR}}_{2}$, ARV, and MF were calculated from the obtained EMG signals and compared with the values derived from the EMG before mixing. Our results suggest the template subtraction method to achieve the best performance among all the methods, providing useful insight for the selection of suitable ECG removal algorithms. In addition, the template subtraction method does not require multichannel recordings nor a reference signal, further increasing its applicability in clinical practice.

## Figures and Tables

**Table 1:** Root mean square error (RMSE) in average rectified value (ARV) and mean frequency (MF) for high-pass filtering (HP) with different cutoffs.

	20 [Hz]	30 [Hz]	40 [Hz]	50 [Hz]	60 [Hz]
ARV [μV]	26.3±8.4	8.8±3.8	5.6 ± 1.8	8.4 ± 2.0	13.4 ± 2.8
MF [Hz]	18.9 ± 4.4	6.1 ± 1.9	13.0 ± 1.5	24.8 ± 2.6	38.9 ± 3.0

**Table 2:** RMSE results for ${\mathrm{KR}}_{2}$, ARV, and MF.

		Data with Healthy ECG					Data with Dysrhythmia ECG				
		MD	$Q_{1}$	$Q_{3}$	LA	UA	MD	$Q_{1}$	$Q_{3}$	LA	UA
${\mathrm{KR}}_{2}$	GT	0.49	0.37	0.81	0.23	1.23	0.55	0.41	0.91	0.26	1.43
	HP	0.11	0.06	0.25	0.00	0.42	0.14	0.08	0.19	0.00	0.29
	TS	0.12	0.08	0.17	0.01	0.27	0.14	0.08	0.18	0.01	0.29
	WT	0.30	0.16	0.43	0.01	0.75	0.16	0.10	0.27	0.02	0.48
	AF	0.12	0.05	0.32	0.00	0.58	0.18	0.06	0.42	0.00	0.93
	ICA	0.10	0.05	0.18	0.01	0.35	0.10	0.05	0.16	0.00	0.32
ARV [μV]	GT	6.6	5.1	8.3	2.8	12.5	5.4	3.6	11.2	1.5	19.5
	HP	7.2	4.0	11.1	1.8	20.6	4.0	2.4	7.2	1.1	11.4
	TS	2.3	1.9	3.0	1.2	4.0	3.8	2.4	7.8	1.7	15.8
	WT	15.8	13.0	20.5	7.5	28.2	11.0	6.2	16.1	3.5	29.4
	AF	7.2	5.4	11.9	2.3	20.4	7.3	4.6	15.8	2.3	32.3
	ICA	3.3	2.4	5.1	1.2	7.8	4.1	2.6	5.7	0.7	8.4
MF [Hz]	GT	5.7	3.6	10.0	1.7	18.5	6.4	4.0	10.7	1.6	19.0
	HP	5.2	4.4	6.8	3.7	9.3	5.7	4.6	6.8	2.9	8.4
	TS	2.0	1.7	2.5	1.1	3.6	3.0	2.2	6.5	1.3	12.7
	WT	6.8	5.8	8.1	4.8	11.1	8.5	7.1	11.2	4.3	17.0
	AF	5.0	3.7	6.1	1.7	9.6	5.4	3.9	8.4	2.0	15.1
	ICA	4.7	2.8	8.4	1.1	16.7	6.1	3.2	9.2	1.5	16.2

MD: median; *Q*_1_: first quartile; *Q*_3_: third quartiles; LA: lower adjacent defined as the smallest value that is equal or larger than *Q*_1_ − 1.5 × (*Q*_3_ − *Q*_1_); UA: upper adjacent defined as the largest value that is equal or smaller than *Q*_3_ + 1.5 × (*Q*_3_ − *Q*_1_).

**Table 3:** Execution time of different methods [ms].

GT	HP	TS	WT	AF	ICA
61.4±3.8	144.2±1.5	274.0±48.2	126.2±5.5	1957.0±769.5	212.4±49.2

## Abbreviations

The following abbreviations are used in this manuscript.

EMG	Electromyogram
ECG	Electrocardiogram
ARV	Average rectified value
MF	Mean frequency
GT	Gating
HP	High-pass filtering
TS	Template subtraction
AF	Adaptive filtering
BSS	Blind source separation
ICA	Independent component analysis
MMC	Máxima Medical Center
MVC	Maximum voluntary contraction
SNR	Signal-to-noise ratio
FIR	Finite impulse response
RMSE	Root mean square error
NLMS	Normalized least mean square
STFT	Short-time Fourier transform
